# The Protective Effect of Uridine in a Rotenone-Induced Model of Parkinson’s Disease: The Role of the Mitochondrial ATP-Dependent Potassium Channel

**DOI:** 10.3390/ijms25137441

**Published:** 2024-07-06

**Authors:** Galina D. Mironova, Alexei A. Mosentsov, Vasilii V. Mironov, Vasilisa P. Medvedeva, Natalia V. Khunderyakova, Lyubov L. Pavlik, Irina B. Mikheeva, Maria I. Shigaeva, Alexey V. Agafonov, Natalya V. Khmil, Natalia V. Belosludtseva

**Affiliations:** Institute of Theoretical and Experimental Biophysics, Russian Academy of Sciences, Pushchino 142290, Russia; makcvel.95@mail.ru (A.A.M.); bceblac@mail.ru (V.V.M.); vasilisa.medv@mail.ru (V.P.M.); nkhunderyakova@gmail.com (N.V.K.); pavlikl@mail.ru (L.L.P.); mikheirinal@yandex.ru (I.B.M.); shigaeva-marija@rambler.ru (M.I.S.); dyneren@gmail.com (A.V.A.); nata.imagination@gmail.com (N.V.B.)

**Keywords:** Parkinson’s disease, rotenone, uridine, mitochondria, mitochondrial ATP-dependent potassium channel, oxidative stress, structure, myeline, neurons

## Abstract

The effect of the modulators of the mitochondrial ATP-dependent potassium channel (mitoK_ATP_) on the structural and biochemical alterations in the substantia nigra and brain tissues was studied in a rat model of Parkinson’s disease induced by rotenone. It was found that, in experimental parkinsonism accompanied by characteristic motor deficits, both neurons and the myelin sheath of nerve fibers in the substantia nigra were affected. Changes in energy and ion exchange in brain mitochondria were also revealed. The nucleoside uridine, which is a source for the synthesis of the mitoK_ATP_ channel opener uridine diphosphate, was able to dose-dependently decrease behavioral disorders and prevent the death of animals, which occurred for about 50% of animals in the model. Uridine prevented disturbances in redox, energy, and ion exchanges in brain mitochondria, and eliminated alterations in their structure and the myelin sheath in the substantia nigra. Cytochemical examination showed that uridine restored the indicators of oxidative phosphorylation and glycolysis in peripheral blood lymphocytes. The specific blocker of the mitoK_ATP_ channel, 5-hydroxydecanoate, eliminated the positive effects of uridine, suggesting that this channel is involved in neuroprotection. Taken together, these findings indicate the promise of using the natural metabolite uridine as a new drug to prevent and, possibly, stop the progression of Parkinson’s disease.

## 1. Introduction

Parkinson’s disease (PD) is a complex neurodegenerative disease, which so far remains incurable. Therefore, all efforts of researchers at present are aimed at the search for substances that prevent and stop this disease [1]. PD, as a rule, is caused by the death of the dopaminergic neurons of the substantia nigra [2]. The depletion of the dopamine pool and a decline in the number of functioning neurons give rise to marked motor, vegetative (metabolic impairment), and mental disorders (psychosis, sleep disturbance, and depression) [3].

A reason for the development of the sporadic form of PD may be environmental factors, including pesticides, herbicides, and neurotoxins such as rotenone, 1-methyl-4-phenyl-1,2,3,6-tetrahydropyridine, and others; some of these cause the disturbance of complex I of the electron transport chain [4]. It is known that any disturbance of the operation of the mitochondrial respiratory chain can entail oxidative stress, as well as the disruption of calcium homeostasis and of tissues: in particular, mitochondria and brain nerve fibers. [5].

One model of PD whose development is accompanied by the manifestation of most motor and nonmotor symptoms, including the appearance of Lewy bodies, is a model induced by the subcutaneous injection of rotenone in an animal [6]. This pesticide inhibits complex I of the mitochondrial respiratory chain, and the inhibition of this very region of the respiratory chain was detected in PD patients [7]. Therefore, rotenone has been widely used for modeling PD. It turns out that the model is one of a few that enables one to observe the maximum broad range of PD symptoms [3].

**Figure 1:**
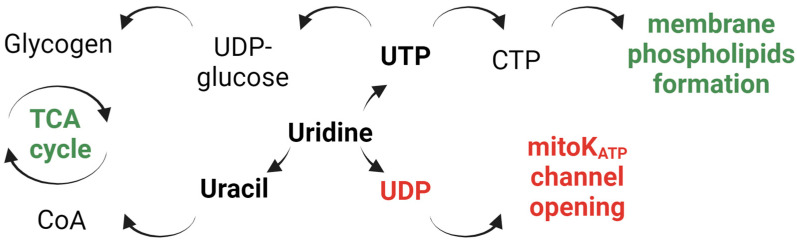
Schematic illustration of the protective pathways of uridine.

Studies on the role of mitochondria in the pathogenesis of PD have shown that a pharmacological activator of the mitochondrial ATP-dependent potassium channel (mitoK_ATP_) contributes to the protection of the organism against the degeneration of dopaminergic neurons [8]. In our laboratory, this channel was isolated for the first time from heart and liver mitochondria and incorporated into the artificial membrane [9]. In addition, we found its metabolic activator, uridine diphosphate (UDP) [10] (Figure 1). UDP by itself is incapable of penetrating the cell [11]; however, its concentration in tissues can increase after the injection of uridine in the animal [12,13].

The goal of the present work was to determine whether uridine can be used as a means for the protection and treatment of PD. It goes through the cell membranes and barriers of the body more easily than other nucleosides [14]. Phosphonucleotides formed from uridine in the cell have pronounced functional properties (Figure 1).

As already noted, UDP is a mitoKATP activator and participates in the protection of tissues against hypoxia [13,15]. Another phosphonucleotide, uridine triphosphate (UTP), is involved through the formation of UDP glucose from it in the synthesis of glycogen [16]. We have recently shown that the administration of uridine has a protective effect in an animal model of diabetes [17]. In addition, uridine, through its decomposition to uracil, is capable of increasing the concentration of acyl CoA, which is necessary for the synthesis of respiratory electron carriers [18]. An increase in the concentration of glycogen and acyl-CoA in tissues improves the functioning of the Krebs cycle and the mitochondria. On the other hand, through exchange with phosphonucleotides, UTP can be converted into cytosine triphosphate (CTP), which is known to be involved in the synthesis of phospholipids and to improve brain function [19].

Taking into account the capability of uridine to normalize energy and oxidative metabolism in the body and to protect brain structures against damage, an attempt was made in this study to develop a new approach to the prevention and treatment of PD using this nucleoside.

## 2. Results

### 2.1. Effect of Uridine on the Physical Condition of Animals with a Model of PD Induced by the Subcutaneous Injection of Rotenone

**Figure 2:**
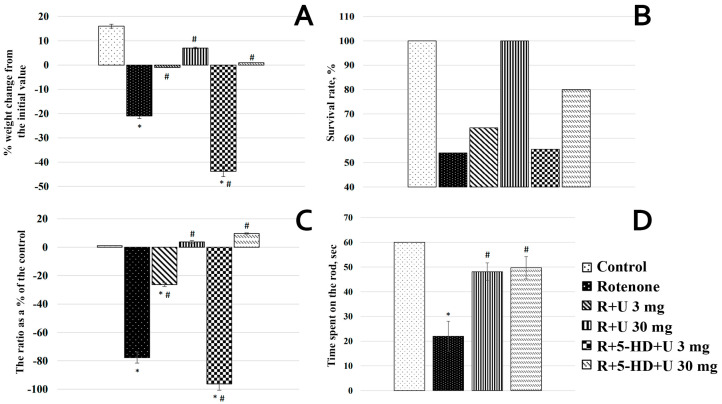
Effect of uridine on the physiological parameters and motor activity of animals with a model of PD induced by the subcutaneous injection of rotenone: (**A**) changes in the weight of animals compared with the initial values; (**B**) survival of animals; (**C**) the open field behavioral test; (**D**) the Rotarod behavioral test. The results are presented as the means ± SEM. * *p* < 0.05 relative to the control; # *p* < 0.05 relative to the rotenone group.

When creating an animal model of PD, first, an optimal concentration of the toxin and the time of its administration to the rats were chosen. The optimal concentration of rotenone was found to be 1.75 mg/kg of body weight. Over the course of the experiment, the weight of animals was monitored by daily weighing. In the control group, an expected increase, with the consideration of age, in the weight of animals was observed, which, by the end of the experiment (28th day) was on average 17% of the initial weight of the animal (Figure 2A). The animals of the rotenone group did not only not gain the weight, but, on the contrary, gradually lost it, beginning from the first injection. By the last weighing, the weight loss was more than 20% of the original value. The injection of uridine at a concentration of 3 mg/kg of body weight diminished the rotenone-induced negative effect by more than 20%, and at a concentration of 30 mg/kg, uridine largely prevented the body weight loss caused by rotenone.

The administration of the mitoK_ATP_ inhibitor 5-hydroxydecanoate (5-HD) in the group with 3 mg rotenone+5-HD+uridine eliminated the positive effect of uridine, bringing it even below the level recorded in the rotenone group. At the same time, in the rotenone+5-HD+uridine 30 mg group, the channel inhibitor, though it eliminated the action of uridine, had less pronounced effects (Figure 2A).

Uridine prevented not only the body weight loss but also the death of animals (Figure 2B). The injection of the toxin in the rotenone group led to 46% mortality. The survival of rats with experimental PD increased after the injection of uridine at a concentration of 3 mg/kg body weight by 10% (from 55% in the rotenone group to 65% in the rotenone+uridine 3 mg group). At the same time, the application of uridine at a concentration of 30 mg/kg in combination with rotenone resulted in 100% survival.

The mitoK_ATP_ inhibitor in the rotenone+5-HD+uridine 3 mg group completely abolished the positive effect of uridine. At a concentration of uridine of 30 mg/kg in the rotenone+5-HD+uridine 30 mg group, the inhibitor eliminated the protective effect of uridine, but not completely (Figure 2B).

### 2.2. Behavioral Changes in Rats with the Rotenone-Induced Model of PD and during the Treatment with Uridine

Because the death of dopaminergic neurons in PD is accompanied by motor disorders, two appropriate behavioral tests with animals were performed. One was an open field test, in which the horizontal motor activity of rats was measured (Figure 2C). As seen from the figure, the motor activity in the rotenone group was reduced, relative to the control, almost by 80%.

The injection of uridine at a concentration of 3 mg/kg in the rotenone+uridine 3 mg group improved the condition of the animal, so that the decrease in the motor activity compared to the control was 25%, which was significantly less than after the administration of rotenone alone. At a concentration of 30 mg/kg, uridine completely abolished the negative effect of rotenone, so that the horizontal motor activity by the 28th day of treatment was close to the control value.

The injection of the specific channel inhibitor in the rotenone+5-HD+uridine 3 mg group eliminated the beneficial effect of uridine and even aggravated the effect caused by rotenone. With uridine at a concentration of 30 mg/kg, no changes in the motor activity relative to the control were observed.

The motor activity of animals was also examined using the Rotarod test. In the control group, no motor impairments were observed; animals freely moved along the rotating rod for 60 s. In separate experiments, it was shown that the rats of the control group could remain on the rod on average for 150 s (2.5 min). However, since the animals of the other groups could stay on the rod for much less than 60 min (Figure 2D), the experiment with the control animals was restricted to 60 s. In the animals of the rotenone group, motor disorders such as bradykinesia and postural instability were observed. They could not stay on the rod for a long time and fell; the time spent on the rod in this group was only 22 s (Figure 2D).

In the rotenone+uridine 3 mg group, no significant changes in this behavioral test compared to the rotenone group were revealed. As can be seen from Figure 2D, the time the animals of the rotenone-uridine 30 mg group spent on the rotating rod was approximately 2.6 times greater than in the rotenone group. The injection of 5-HD in the animals in the rotenone+uridine 30 mg group did not significantly affect this parameter.

### 2.3. Effect of Uridine on the Functioning of Mitochondria from the Cerebral Cortex in the Model of PD Induced by the Administration of Rotenone

It is known that one of the reasons for the mitochondrial dysfunction that develops in PD is the impairment of the functioning of the respiratory chain complexes, and, in particular complex I, which leads to an increase in the production of reactive oxygen species (ROS) [7]. In the case that the antioxidant systems do not cope with increased ROS formation, oxidative stress develops in tissues. An increase in ROS production in the substantia nigra of the brain leads to the oxidation of dopamine and the death of neurons [20]. Therefore, one of the goals of the present study was to measure the rate of hydrogen peroxide formation in the brain cortex (BC) mitochondria in the PD model and to determine whether this characteristic can be improved by uridine. It follows from the literature that about 80% of peroxides in the body are synthesized in mitochondria [21].

**Figure 3:**
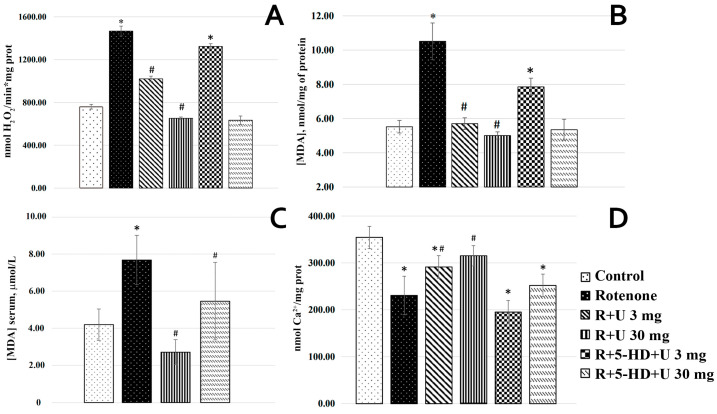
Effect of uridine on the biochemical parameters in mitochondria from the brain cortex and blood serum of animals with a rotenone-induced model of PD: (**A**) the rate of hydrogen peroxide formation in mitochondria; (**B**) the production of MDA in mitochondria; (**C**) the production of MDA in blood serum; and (**D**) the calcium retention capacity of mitochondria. Respiration substrates: 5 mM malate and 2.5 mM glutamate. The results are presented as the mean ± SEM. * *p* < 0.05 relative to the control, # *p* < 0.05 relative to the rotenone group.

As seen from Figure 3A, rotenone increased the rate of hydrogen peroxide formation in BC mitochondria by almost twofold compared with that in the control group. The injection of uridine at a concentration of 3 mg/kg decreased this parameter by 30%, and at a concentration of 30 mg/kg, uridine almost completely restored it to the control values. The mitoK_ATP_ inhibitor in the group with uridine at a concentration of 3 mg/kg almost completely eliminated the effect of uridine, whereas at a concentration of 30 mg/kg, the protective action of uridine was hardly affected by 5-HD.

An increase in the rate of hydrogen peroxide formation led to the accumulation of lipid peroxides in mitochondria, which was determined from the concentration of malondialdehyde (MDA). In the mitochondria of the BC of animals with experimental parkinsonism, the MDA concentration increased almost by 90% compared to the control (Figure 3B). The injection of uridine at a concentration of 3 mg/kg and 30 mg/kg decreased the MDA concentration to the control values. The administration of 5-HD abolished the positive effect of uridine, increasing the MDA concentration by almost 40% in the rotenone+5-HD+uridine 3 mg group. At a concentration of 30 mg/kg in the rotenone+5-HD+uridine 30 group, the positive effect of uridine was retained.

It was found that the concentration of lipid peroxides in the PD model changed not only in rat brain tissues but also in blood serum. As is evident from Figure 3C, the MDA concentration in blood serum from rats with PD is twofold higher compared to the control. It was found that uridine at a concentration of 30 mg/kg significantly lowered the MDA concentration in rats with PD, and the inhibitor 5-HD neutralized, though not completely, the effect of uridine.

With the development of oxidative stress in mitochondria, the disturbance of calcium homeostasis occurs, which is determined by measuring the mitochondrial calcium retention capacity. It was found that the injection of rotenone in an animal decreased the calcium retention capacity of mitochondria by more than twofold (Figure 3D). Uridine at a concentration of 3 mg/kg prevented this decrease, and the combined application with the mitoK_ATP_ inhibitor partially abolished the protective effect of uridine. With uridine at a concentration of 30 mg/kg, similar effects of the mitoK_ATP_ modulators on the mitochondrial retention capacity were observed.

### 2.4. Effect of Uridine on the Functioning of Mitochondria from the Cerebral Cortex in the Model of PD Induced by the Administration of Rotenone

Since the cause of PD is the depletion of the dopamine pool and the death of cortical neurons [3], we studied the structure of the substantia nigra neurons in the rotenone-induced PD model before and after the correction of the disease with uridine.

**Figure 4:**
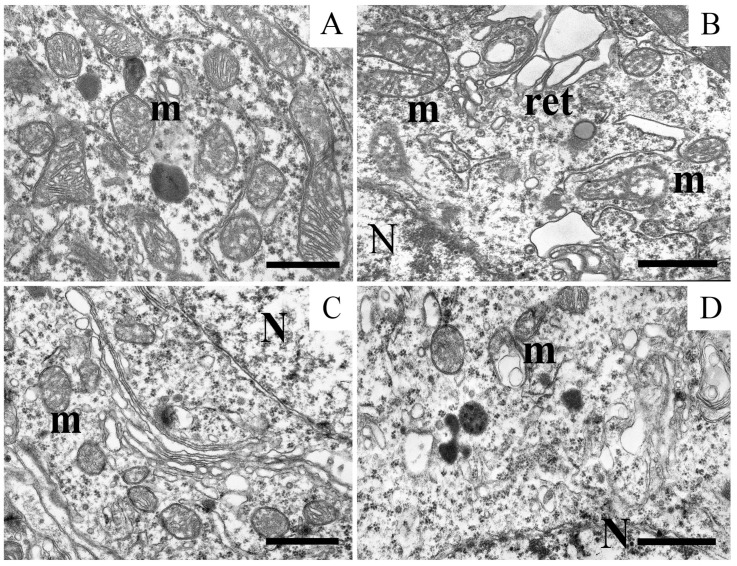
Part of the soma of a dopaminergic neuron of the substantia nigra in the control preparation (**A**) with intact mitochondria; and (**B**) mitochondria with the cytoplasmic reticulum loops and disrupted cristae structure in a model of parkinsonism after treatment with rotenone (**B**). (**C**) Recovery of the mitochondrial structure in the presence of uridine. (**D**) Disturbed mitochondria in the presence of uridine and 5-HD. Scale bar: 1 µm, m—mitochondria, N—nucleus, and ret—reticulum.

An ultrastructural examination showed that the neurons of the substantia nigra of the rat brain in control samples had a structure typical of central neurons. The nucleus was surrounded by a spherical envelope with the nucleolus located in the center. The cytoplasm contained a network of flattened cisternae of the granular endoplasmic reticulum and of the Golgi complex. Lysosomes of various shapes were also found. Mitochondria showed a well-preserved membrane structure and densely packed cristae (Figure 4A).

Modeling PD with rotenone led to significant damage to the intracellular structure of neurons. The outer membrane of the nucleus envelope stretched and formed loops. The nucleolus in the nucleus was located near the membrane. The cisternae of the Golgi complex located in the cytoplasm and of the network of the granular endoplasmic reticulum were dilated. Mitochondria in the soma of neurons in the experimental animals underwent significant structural changes. They showed different degrees of destruction; swollen organelles with almost destroyed cristae and mitochondria with ribbon-shaped cristae were encountered. The loss of membrane integrity and the lysis of mitochondrial cristae were also observed (Figure 4B).

**Figure 5:**
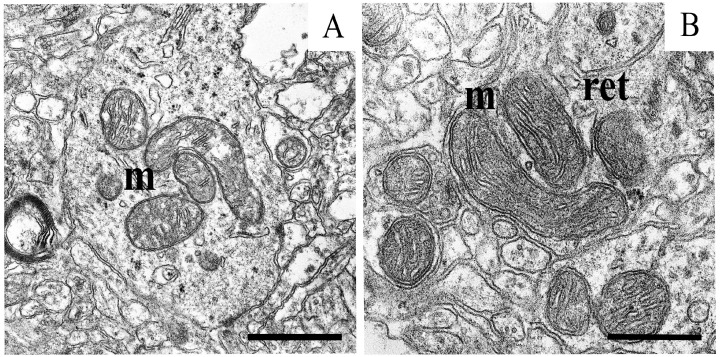
Mitochondria in the soma of a neuron of the substantia nigra after the injection of 3 mg (**A**) and 30 mg of uridine (**B**). Scale bar 1 µm. m—mitochondria; ret—reticulum.

It was found that uridine protects the structure of the neurons of the substantia nigra in a dose-dependent manner, restoring the integrity of mitochondria and the myelin sheaths. At a concentration of 3 mg/kg, it markedly improved the neuron structure (Figure 4C and Figure 5A). The nuclear envelope returned to its spherical shape, typical of control samples. The structure of the Golgi complex was also close to the intact form. The cisternae of the granular endoplasmic reticulum acquired a flattened appearance. Liposomes and lipofuscin granules were as before, when we had encountered them in the cytoplasm. At a concentration of 30 mg/kg, uridine completely restored the structure of neurons (Figure 5B), and the picture was similar than that in the control group.

The ultrastructural analysis also showed that the combined action of rotenone, uridine, and 5-HD eliminates the effect of the structural restoration caused by uridine (Figure 4D). After the addition of 5-HD, the ultrastructure of intracellular organelles destroyed by the action of rotenone and restored after the injection of uridine was similar to what was observed after the administration of rotenone alone. The dilated cisternae of the Golgi complex and destroyed mitochondria with the partial lysis of cristae were seen.

In the neurons of the substantia nigra, the stratification of the myelin sheath was also observed (Figure 5B), which usually did not occur in the control. The sheath lost its inherent structure and consisted of separate lamellae, acquiring bizarre shapes.

**Figure 6:**
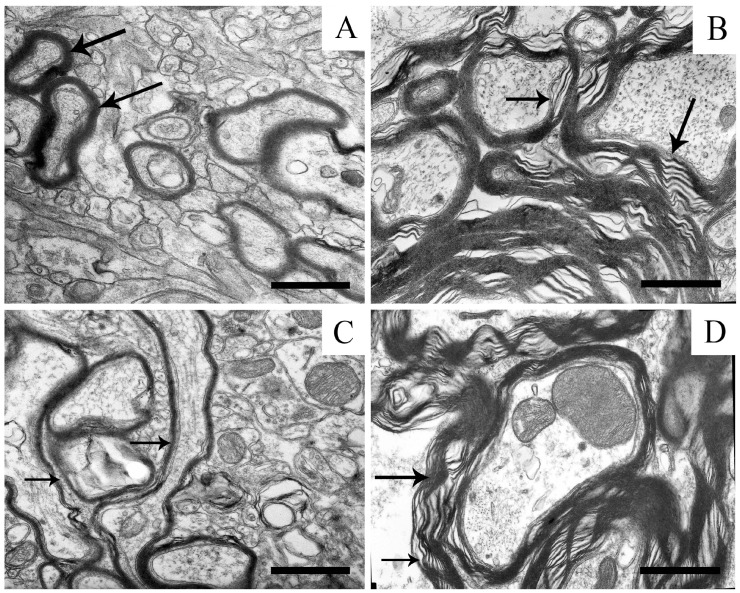
Myelin sheaths of the axons of the neuropil of the substantia nigra in the control (**A**) with densely packed membrane layers (arrows); (**B**) unwinding of the lamellae of the myelin sheath (arrows) after treatment with rotenone in a model of parkinsonism; (**C**) restoration of the myelin structure (arrows) after the action of uridine; (**D**) disruption myelin structures (arrows) after the combined action of uridine and 5-HD. Scale bar: 1 µm.

In the presence of uridine at a concentration of 3 mg/kg, the myelin sheath acquired its usual shape and represented the multitude of densely packed membrane layers (Figure 6C). The mitochondria had a well-preserved structure with densely packed cristae. Uridine, at a concentration of 30 mg/kg, completely eliminated the disturbances in the substantia nigra induced by rotenone, and 5-HD only insignificantly affected the action of uridine under these conditions.

### 2.5. Effect of Uridine on the Cytobiochemical Characteristics of Energy Metabolism in Blood Lymphocytes of Animals with the Rotenone-Induced PD Model

The cytobiochemical determination of the activity of succinate dehydrogenase (SDH) and lactate dehydrogenase (LDH) in blood lymphocytes can be used as a sensitive method for characterizing the energy balance (aerobic and anaerobic exchange) in animals and humans in clinical and laboratory diagnoses, as well as for monitoring treatment progress [22].

**Figure 7:**
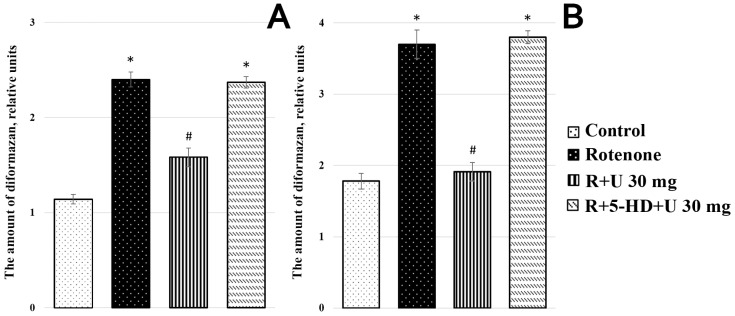
The activity of the enzymes LDH (**A**) and SDH (**B**) in lymphocytes from rat blood in experimental groups. The incubation medium contained 125 mM KCl, 10 mM HEPES, and 1.22 mM nitro blue tetrazolium (NBT) (pH 7.2) and was supplemented with 5 mM potassium lactate (in the presence of 5 mM potassium malonate and 0.5 mM NAD) for determining the LDH activity, or 5 mM succinic acid for determining the activity of SDH. Designations: U, uridine; R, rotenone. The results are presented as the mean ± SEM. * *p* < 0.05 relative to the control, # *p* < 0.05 relative to the rotenone group.

The administration of rotenone into rats to model PD led to a significant (*p* ≤ 0.05) change in the activity of SDH by two times and a more than twofold increase in the activity of LDH (*p* ≤ 0.01) (Figure 7). It is seen that uridine at a concentration of 30 mg/kg body weight decreased the hyperactivation of SDH and LDH in sick rats to values close to the control group, and the mitoK_ATP_ inhibitor 5-HD abolished the positive effect of uridine.

## 3. Discussion

In this work, we studied the protective effect of uridine in a rotenone-induced PD model in rats with impairments of the motor function and biochemical characteristics in the brain tissue and blood serum, as well as with structural alterations in the substantia nigra. In addition, the role of the mitoK_ATP_ channel in the pathogenesis of PD was elucidated using the inhibitory analysis.

The study of the motor activity in model animals showed that the administration of rotenone caused its marked decline, which was partially eliminated by the injection of uridine at a concentration of 3 mg/kg and was almost completely restored at a uridine concentration of 30 mg/kg. Similar improvements in motor function in PD were observed when uridine was used in combination with docosahexaenoic acid [23,24]. We have found previously that uridine increases the concentration of the mitoK_ATP_ channel opener in tissues, specifically, uridine diphosphate [12,13]. We believe that the restoration of the motor activity of animals by uridine is associated with the activation of this channel. This is supported by the fact that the positive effect of uridine at a concentration of 3 mg/kg of body weight was abolished by the specific inhibitor of this channel 5-HD (Figure 4). However, at a concentration of 30 mg/kg, the channel inhibitor produced a weaker effect. Presumably, the restoration of the motor activity of animals after the injection of 30 mg/kg of uridine is associated not only with the activation of the channel, but also with an increase in energy metabolism in mitochondria. This assumption follows from the known potential of uridine to affect multiple targets. In the body of an animal, it can decompose to uracil, thereby increasing the concentration of uracil CoA, which is necessary for the operation of the Krebs cycle and the synthesis of reducing equivalents in the respiratory chain [18]. At the same time, after the administration of 30 mg/kg of uridine, along with UDP, UTP forms in tissues [16], which participates in the synthesis of glycogen, a source of substrates for the work of the respiratory chain. The assumption that uridine enhances energy metabolism in animals is also confirmed in this work by the measurements of other parameters.

It was found that uridine normalized the physiological characteristics of rats that changed after modeling the pathology. It dose-dependently restored the animal weight lost after the injection of rotenone. Of special note is the finding that uridine was capable of preventing the mortality of animals, which was on average 46% in this model. At a uridine concentration of 3 mg/kg of body weight, mortality was reduced to 36%, and at a concentration of 30 mg/kg, uridine completely prevented the death of animals. Because the inhibitor of the mitoK_ATP_ channel in the rotenone+5-HD+uridine 3 mg group completely abolished the beneficial effect of uridine, we assumed that the protective effect of uridine at a concentration of 3 mg/kg is largely associated with the opening of this channel, which, under physiological conditions, is closed. With uridine at a concentration of 30 mg/kg, the mitoK_ATP_ inhibitor had a much weaker effect. Probably, in the rotenone-induced PD model, uridine at this concentration prevents the development of the pathology, not only due to the activation of the channel, but also by restoring the energy metabolism in experimental rats. It should be emphasized that uridine at a concentration of 30 mg/kg was found to prevent the development of mitochondrial dysfunction in the heart tissue in hypoxia [13], suppress inflammatory processes in the whole body [12], increase its endurance during intensive physical activity, and inhibit the development of PD induced by the neurotoxin 6-OHDA [8].

It is worth noting that the 6-OHDA model differs from the model used in the present work, in that the neurotoxin is injected once immediately into the substantia nigra of the brain, where it initiates the onset of the early stage of neurodegeneration in PD [25]. In the case of the rotenone-induced PD model, the complex I inhibitor rotenone is injected subcutaneously (into the withers of the animal) for a long period of time; owing to its high lipophilicity, it readily penetrates cell membranes and barriers of the body and induces multiple signs of PD in animals [26]. Presumably, this is the reason for a higher mortality observed in animals with this model. Rotenone is known to inhibit the work of the complex I respiratory chain, which has been observed in patients [7]. In this case, it induces the development of not only oxidative stress but also mitochondrial dysfunction, whereas 6-OHDA induces mainly the development of oxidative stress, as a result of an increase in the concentration of ROS in tissues [27]. Therefore, the disturbances we have observed when modeling PD using 6-OHDA are similar to those in the rotenone model, but they are less pronounced and are almost completely inhibited by the mitoK_ATP_ channel opener [8].

It has been previously found that the activation of the mitoK_ATP_ channel protects the body against hypoxia, and the inhibitor of the channel eliminates the positive effect of hypoxic preconditioning [28]. The mechanism of this phenomenon is as yet unclear. We suppose that the opening of the channel results in the restoration of the redox metabolism in hypoxia through a decline in the formation of ROS, which usually accumulate in abnormal amounts in this condition. It is known that, during hypoxia, a state of hyper-restoration in respiratory chain electron carriers is observed, which leads to an increase in the rate of the formation and accumulation of abnormal amounts of hydrogen peroxide in the respiration chain [29]. The opening of this channel increases the rate of entry of potassium into mitochondria and activates the potassium cycle [30], which lowers the mitochondrial membrane potential (ΔΨm) and prevents the excessive restoration of respiratory electron carriers [31]. A decrease in ΔΨm has found been previously after the addition of the pharmacological activator of the mitoK_ATP_ channel diazoxide [32].

In this work, we showed in the mitochondria of the BC of rats with experimental parkinsonism that uridine has a beneficial effect on the characteristics of oxidative and ion metabolism. Thus, at a concentration of 3 mg/kg, it significantly reduced a twofold increase in the rate of H_2_O_2_ formation in rats injected with rotenone, and at a concentration of 30 mg/kg, completely prevented it. The inhibitor of the mitoK_ATP_ channel 5-HD completely abolished the protective effect of the nucleoside. This supports the protective role of the channel in preventing the rotenone-induced pathology.

An increase in the rate of H_2_O_2_ formation naturally leads to an increase in the concentration of lipid peroxides, which is also removed by low concentrations of uridine. The protective effect in this case is associated with channel activation. Consequently, a uridine concentration of 3 mg/kg is sufficient to normalize the oxidative metabolism in the mitochondria of animals in this PD model. A uridine concentration of 30 mg/kg so effectively prevents the development of hypoxia and oxidative stress that the protective role of the mitoK_ATP_ channel in normalizing the oxidative metabolism manifests itself to a lesser extent.

The fact that similar pathological changes in oxidative metabolism, namely, in the level of MDA, were found not only in brain tissues but also in blood serum indicates that oxidative stress in the animal PD model develops in the whole body. As shown by the inhibitory analysis in the mitochondria of blood lymphocytes, in which the mitoK_ATP_ channel has also been identified [33], the protective effect of uridine is associated with the functioning of this channel.

It is known that PD develops most intensively during aging, when the rate of ROS formation increases [34]. We have shown previously that the activity of the mitoK_ATP_ channel in an aging organism decreases significantly, namely by three to five times. In this case, no significant changes in the functioning of the respiratory chain were observed [35]. The fact that PD develops in the body when the activity of the channel decreases confirms the data obtained in this work on the role of the channel in the pathogenesis of PD and, probably, of other neurodegenerative diseases that usually develop during aging.

In this work, changes were also found in the calcium metabolism in mitochondria from the BC, which appeared to show a decrease in their capacity to retain this ion, thereby facilitating the opening of the calcium-dependent cyclosporin A-sensitive pore, which may lead to cell death [36]. That uridine restores this ability, and that the mitoK_ATP_ inhibitor prevents its protective effect, confirms the assumption that the channel plays a role in the regulation of the mitochondrial pore and, consequently, cell death. This agrees with the data obtained in this work on the prevention of the death of animals after the injection of uridine.

The data obtained in this study indicated that the disturbance of mitochondrial functions occurs not only in the BC but also in the lymphocytes of animals with experimental parkinsonism. The cytochemical examination revealed a significant increase in the activity of both SDH, an enzyme involved in aerobic metabolism, and LDH, a key enzyme in glycolysis, indicating the impairment of energy metabolism in lymphocytes. The fact that uridine normalizes these characteristics to the control level, and that 5-HD completely eliminates the protective effect of uridine, confirms the involvement of the mitoK_ATP_ channel in the general pathogenesis of PD.

The enhancement of SDH activity found in animals with model parkinsonism may be explained by the activation of transamination processes, which are closely related to cytosol alanine transaminase; as a result, the metabolism switches toward the predominant oxidation of succinate, a mediator of the sympathetic nervous system [37]. This state of SDH is called stressful or hyperactive [38,39]. Indeed, as indicated by our recent data, under hypoxia, an increase in the succinate concentration and the SDH activity, as well as an increase in the expression of the enzymes of complex II of the respiratory chain and a decrease in the expression of complex I, are observed in the brain tissue [40]. In turn, the increase in LDH activity in the lymphocytes of model animals under hypoxia can be explained by a reduction in the amount of oxygen present. In this case, an adaptive increase in glycolysis occurs. It should be noted that the rate of glycolysis, in this case, despite its lower efficiency, is considerably higher than that of oxidative phosphorylation [41].

That uridine reduces oxidative stress and normalizes glycolysis and oxidative phosphorylation indicates that it has an antihypoxic effect on processes occurring not only in tissues, but also in lymphocytes. These findings show promise for the application of the method of the cytochemical determination of SDH and LDH activity, recently developed in our laboratory as a sensitive method for characterizing the energetic state in animals and humans in clinical and laboratory diagnostics, as well as for monitoring treatment progress [22,42].

In animals with rotenone-induced parkinsonism, structural alterations in the neurons of the substantia nigra of the brain were also observed, which affected the state of cisternae of the endoplasmic reticulum and mitochondria. In the substantia nigra, we detected giant vacuoles with the expansion of the perinuclear space toward the cytoplasm. It is known that the formation of these vacuoles can result from a decrease in the activity of the proteasome system, which is also involved in the pathogenesis of many neurodegenerative diseases, including PD [43].

A partial lysis of the cristae detected in the study points to a reduction in the energy efficiency of mitochondria and a deficit of ATP required for the normal functioning of neurons. The accumulation of a large number of lipofuscin granules in the soma of the neuron usually occurs on normal aging and in neurodegenerative diseases. Therefore, the intensive accumulation of lipofuscin in the soma of neurons in our experiments indicates the degeneration process in neurons.

The electron microscopy examination also revealed the destruction of the myelin sheath of axons, which serves to isolate nerves from one another and accelerate the transport of signals along long nerves. Without these functions, the signals become mixed, and normal movements become impossible. Some degenerative diseases, such as multiple sclerosis, in which antibodies to myelin are present in the patient’s blood, also lead to the degradation of the myelin sheath [44,45]. This may give rise to a great number of side-effects and, in the final analysis, to complete paralysis and death.

The mechanism of the protective effect of uridine in this case is related to its ability to form UTP, which, through the exchange of nucleotides, can be converted to CTP [13,19]. In turn, the maintenance of the concentration of CTP in brain cells is a necessary condition for the synthesis of phospholipids and the functioning of membrane components, such as myelin, which we have recently shown in a model of PD induced by the neurotoxin 6-hydroxydopamine [8]. An increase in the concentration of CTP in tissues after the injection of uridine stimulates the synthesis of brain phospholipids, which probably prevents the destruction of the myelin sheath.

To summarize, this study on a model of rotenone-induced PD confirmed the manifold protective effect of uridine. As shown by the inhibitory analysis, uridine at low concentrations (3 mg/kg of body weight) is capable of activating the mitoK_ATP_ channel. This leads to a decline in the severity of disturbances of oxidative and ion metabolism in the mitochondria of brain tissues and the prevention of the opening of the calcium-dependent nonselective mitochondrial pore. Under these conditions, the motor activity of rats is partially restored as a result of the prevention of significant injuries in the neuron structure and the myelin sheath of nerve fibers, and the mortality of animals decreases. Increasing the uridine concentration to 30 mg/kg leads to an almost complete restoration of oxidative, ion, and energy metabolism, as well as the restructure of neurons and the myelin sheath, which prevents the death of animals.

Recent preclinical and clinical studies have shown that synthetic activators of the mitoK_ATP_ channel [46], in particular, sildenafil, can be used for the treatment of neurodegenerative pathologies, including Parkinson’s and Alzheimer’s diseases [47,48]. The results of phase-2 clinical trials have demonstrated that sildenafil is effective in reducing dyskinesia in patients with Parkinson’s disease [NCT02162979]. However, the use of this drug has limitations for elderly patients with comorbidities of the cardiovascular system, and its dose must be strictly controlled [47]. It should be noted that natural channel activators such as uridine have advantages over synthetic drugs, owing to their lack of undesirable side effects.

Taken together, these findings suggest that the natural metabolite uridine is a promising drug for preventing and, possibly, stopping the progression of PD.

## 4. Materials and Methods

Mature Wistar male rats weighing 230–260 g were used. This study was performed in accordance with the “Rules for conducting research with experimental animals” (Order of the Ministry of Health of Russia, 12 August 1997, no. 755). All manipulations with rats were carried out in compliance with the Directions of the European Parliament 2010/63/EC and were approved by the Commission on Biosafety and Bioethics (Institute of Theoretical and Experimental Biophysics, Russian Academy of Sciences; Permission no. 8 of 8 February 2023).

### 4.1. Administration of the Neurotoxin Rotenone and Uridine to Animals in an Experimental Model of Parkinson’s Disease

Animals were arbitrarily divided into six groups and were maintained under the same temperature and humidity conditions with free access to standardized food and water (Table 1).

For modeling the disease, rotenone at a concentration of 1.75 mg/kg of body weight was used. The rotenone model was reproduced by the chronic subcutaneous injection of the herbicide dissolved in a DMSO–Intralipid mixture (volume ratio 2: 98%) to the interscapular zone of an animal. The modeling was performed using the following scheme: injections for 2 days/2 days break over a period of 28 days. Fifteen minutes after the administration of rotenone, the activator of the mitoK_ATP_ channel uridine (PanReac AppliChem, Darmstadt, Germany) was injected intraperitoneally at a concentration of 3 or 30 mg/kg of body weight, according to the scheme: injections for 2 days/2 days break for 28 days. Fifteen minutes after the administration of uridine, the inhibitor of the mitoK_ATP_ channel 5-hydroxydecanoate was injected intraperitoneally at a dose of 5 mg/kg of body weight, according to the same scheme.

### 4.2. Isolation of Mitochondria from the Cerebral Cortex of the Rat

On the 28th day of the experiment, the rats were decapitated and the brain tissue was washed in physiological saline, rapidly placed in an isolation medium (0.075 M saccharose, 0.225 M mannitol, 10 mM HEPES-KOH, 1 mM EDTA, pH 7.4), and crushed. The brain tissue was homogenized using a manual homogenizer and centrifuged at 2000× *g* for 5 min. The supernatant was centrifuged at 12,000× *g* for 10 min. The residue was added to the washing medium (0.075 M saccharose, 0.225 M mannitol, 10 mM HEPES-KOH, pH 7.4), homogenized, and centrifuged at 12,000× *g* for 10 min. The resulting sediment was homogenized in the washing medium. All procedures were carried out on ice in a cold room at a temperature of +4 °C.

### 4.3. Behavioral Tests

At present, the only noninvasive method for identifying PD is the assessment of motor disorders. In our study, the symptoms of the disease were revealed using behavioral tests and a visual examination of the condition of the animal.

In the open field test, the horizontal motor activity of the animals was recorded, which included running along different trajectories and circling around one place. The main criterion for characterizing this form of activity is the participation of all four paws in the movement of the animal. The animal was placed in a box 80 × 80 cm in size with a wall height of 40 cm. The bottom of the box was divided into squares 10 × 10 cm in size. The animal could freely move in this field. All squares crossed by the animal were counted; the test was conducted for 10 min, and the parameters of motor activity in experimental and control animals were estimated.

The effect of drugs on motor coordination and endurance in rodents is estimated using the Rotarod test [49]. This testing is carried out using a special apparatus; the procedure by itself is safe and humane. Animals are placed on a rotating drum; the surface of the drum is textured to prevent their slipping. When an animal falls down onto an individual sensor platform, the results of the test are displayed on the front panel of the apparatus. In our test, a constant speed of drum rotation of 8 cm/s was used, and the animals stayed on the rod for 60 s, after which the test was terminated. Testing was carried out three times in one day to determine the mean time the animal spent on the rotating rod.

### 4.4. Determination of the Ca^2+^ Retention Capacity of Brain Mitochondria

The Ca^2+^ retention capacity of rat brain mitochondria was determined using a Ca^2+^-selective microelectrode (NikoAnalit, Moscow, Russia) and a Record4 electrometric device (ITEB RAS, Pushchino, Russia). The incubation medium contained 150 mM KCl, 1 mM NaH_2_PO_4_, 10 µM EGTA, and 10 mM HEPES-KOH (pH 7.4). The concentration of the mitochondrial protein in a cuvette was 0.4–0.5 mg/mL. The following substrates were used: 5 mM malate + 5 mM potassium glutamate. CaCl_2_ was added in fractions of 25 µM every 30 s. The calcium retention capacity was calculated as nmol Ca^2+^/mg protein.

### 4.5. Determination of the Rate of Hydrogen Peroxide Formation in Brain Mitochondria

The generation of hydrogen peroxide by rat brain mitochondria was measured using a fluorimeter (Varian Medical Systems, Palo Alto, CA, USA) with the excitation set at 570 nm and the emission at 585 nm. The reaction mixture contained 120 mM KCl, 5 mM KH_2_PO_4_, 10 mM HEPES-KOH, and 0.5 mM EGTA, at pH 7.4. The production of hydrogen peroxide by brain mitochondria was measured at 37 °C in the presence of 20 µM Amplex Red and 2 U/mL horse radish peroxidase. To avoid the influence of the optical effects associated with mitochondrial swelling, mitochondrial activity was measured in the dual-wave registration mode (572–600 nm) [50]. To more precisely estimate the rate of peroxide formation in brain mitochondria, the inhibitor of catalase 10 mM 3-amino-1,2,4-triazole (Sigma–Aldrich, St. Louis, MO, USA) was added to samples. The concentration of mitochondria in the incubation medium was 0.2 mg/mL. The rate was expressed in nmol H_2_O_2_/min × mg protein.

### 4.6. Determination of Lipid Peroxidation Products in Brain Mitochondria and Blood Serum

The concentration of lipid peroxidation products in the brain mitochondria and blood serum of rats was determined from the reaction of 250–275 µL of 5% SDS, 900 µL of 20% CH_3_COONa, and 300 µL of a 0.8% solution of thiobarbituric acid (TBA), and 50–25 µL of the blood serum or mitochondria. Eppendorf plates with the reaction mixture were incubated for 1 h at 90 °C. Then, the liquid was centrifuged at 2000× *g* for 5 min. The product was a trimethine complex of a characteristic pink color (λ max = 532 nm). Measurements were performed on a spectrophotometer (Shimadzu Europa GmbH, Kyoto, Japan) at a wavelength of 532–650 nm. The results were expressed as nmol/min × mg protein.

### 4.7. Determination of the Activity of Succinate Dehydrogenase and Lactate Dehydrogenase in Immobilized Lymphocytes on a Blood Smear

The activity of two key dehydrogenases in blood lymphocytes was determined from the reduction of nitro blue tetrazolium to diformazan using a cytobiochemical method [22]. The activity of SDH was determined by adding a substrate (5 mM succinic acid), and the activity of LDH was determined in the presence of 5 mM lactic acid, 5 mM malonate, and 1 mM nicotinamide adenine dinucleotide (NAD) in the incubation medium containing 125 mM KC1, 10 mM HEPES, and 1 mg/mL of nitro blue tetrazolium, pH 7.2 ± 0.05. After fixation with 60% acetone for 30 s, blood smears were incubated with substrates at 37 °C for 60 min, after which nuclei were additionally stained for a better visualization with 0.05% neutral red for 8 min. The microscopic examination of blood smears was performed under a Leika M 2000 microscope, taking no less than 100 microphotographs from each smear at a magnification of 100 × 10 under oil emission. A cytomorphological, quantitative analysis of the color microphotographs of lymphocytes was performed using the programs Bloodrunner and Cell Composer, which make it possible to determine the amount and distribution of the dye diformazan in each cell. The average area stained by formazan (in µM^2^) in a sample of 100 lymphocytes in each animal was calculated.

### 4.8. Electron Microscopic Examination of Slices of the Substantia Nigra of the Brain

After decapitation, pieces of the substantia nigra were taken from the rat brain and fixed in a 2.5% solution of glutaraldehyde in a 0.1 M cacodylate buffer (pH 7.4). Postfixation was carried out with a 2% solution of osmium tetroxide in a 0.1 M cacodylate buffer (pH 7.4). Dehydration was conducted in alcohols of increasing concentration and acetone. Dehydrated samples were embedded in Epon 812 resin. Ultrathin sections 60–70 nm thick were prepared on a Leica EM UC7 microtome (Wetzlar, Germany). Ultrathin sections were contrasted with 1% uranyl acetate and lead citrate. The samples were scanned using a JEOL JEM-100B electron microscope (Tokyo, Japan).

### 4.9. Statistical Analysis

All values are given as the means ± standard error of the means. Statistical analysis was performed using a one-way analysis of variance (ANOVA) to assess the overall significance among experimental groups. When differences were significant, multiple comparisons were performed using Tukey’s multiple comparisons test. MS Excel 2021, ImageJ 1.8.0., and Prism GraphPad 7 (GraphPad Software, RRID: SCR_002798) software programs were used for the data and statistical analysis. Differences are significant at *p* < 0.05.

## 5. Conclusions

The data obtained in the study show promise for applying the natural metabolite uridine as a novel drug to prevent and, probably, stop the development of PD. The mechanism of the neuroprotective action of uridine may be related, among other things, to its ability to activate the mitochondrial mitoK_ATP_ channel and prevent the progression of mitochondrial dysfunction in the brain tissue.

## Figures and Tables

**Table 1:** Experimental groups of rats.

Experimental Groups	Injections			
	DMSO-Intalipid Mixture, mL/kg	Rotenone, mg/kg	5-HD, mg/kg	Uridine, mg/kg
Control (*n* = 32)	1.5	-	-	-
Rotenone (*n* = 43)	-	1.75	-	-
Rotenone + uridine 3 mg (*n* = 26)	-	1.75	-	3.00
Rotenone + uridine 30 mg (*n* = 21)	-	1.75	-	30.00
Rotenone +5-HD + uridine 3 mg (*n* = 28)	-	1.75	3.00	3.00
Rotenone +5-HD + uridine 30 mg (*n* = 21)	-	1.75	3.00	30.00

## Data Availability

The data presented in this study are available on request from the corresponding author.
